# Lower body negative pressure enhances oxygen availability in the knee extensor muscles during intense resistive exercise in supine position

**DOI:** 10.1007/s00421-019-04113-w

**Published:** 2019-03-26

**Authors:** Dajana Parganlija, Vita Nieberg, Marc Sauer, Jörn Rittweger, Wilhelm Bloch, Jochen Zange

**Affiliations:** 1 Space Physiology Department, Institute of Aerospace Medicine, German Aerospace Center (DLR), Cologne, Germany; 2 Institute of Cardiovascular Research and Sports Medicine, German Sport University, Cologne, Germany; 3 Present Address: IQWiG, Institute for Quality and Efficiency in Health Care, Im Mediapark 8, 50670 Cologne, Germany; 4 Department of Sport and Health Sciences, Technical University of Munich, Munich, Germany; 5 Institute of Training Science and Sport Informatics, German Sport University Cologne, Cologne, Germany; 6 Department of Pediatrics and Adolescent Medicine, University of Cologne, Cologne, Germany

**Keywords:** Slow concentric–eccentric resistance exercise, Lower body negative pressure, Simulated orthostasis, Muscle perfusion, Muscle pump, Leg press

## Abstract

**Purpose:**

During exercise in supine posture or under microgravity in space, the gravity-dependent component of local blood pressure in leg muscles at upright posture can be simulated by lower body negative pressure (LBNP). We hypothesized that during resistive exercise LBNP favors oxygen availability in lower extremities, benefiting energy levels and performance of working muscles.

**Methods:**

In permutated crossover design, nine subjects performed a series of fifteen slow-paced concentric (4 s) and eccentric contractions (4 s) without or with 40 mmHg LBNP and 4 s pause between repetitions. The force at knee flexion was 6% of the one repetition maximum (1-RM) and gradually increased to 60% 1RM in the first half of the individual range of motion, subsequently remaining constant until full extension.

**Results:**

During the low force periods of continuous exercise, LBNP enhanced the refill of capillary blood measured by near infrared spectroscopy, amplifying the increase of total haemoglobin by about 20 µmol/l (*p* < 0.01) and oxyhaemoglobin by about 10 µmol/l (*p* < 0.01). During continuous exercise, LBNP induced a trend towards a lower EMG increment. This LBNP effect was not found when the periods of low forces at knee flexion were extended by 4 s pauses. Increased respiratory oxygen uptake (+ 0.1 l/min, *p* < 0.05) indicated overall enhanced muscle energy turn-over.

**Conclusions:**

Our results suggest stimulation of oxidative metabolism through LBNP enables working muscles to meet the energy demands of intense exercise. Further research is needed on the consequences for energy metabolism and the molecular control of growth and differentiation.

## Introduction

In upright body posture, perfusion of working leg muscles profits from the muscle pump, which increases the arterio-venous difference of blood pressure. The mechanism involves a gravity-dependent pressure component on the arterial side and a reduction of pressure on the venous side by expulsion of blood and prevention of backflow through venous valves (Laughlin and Schrage 1999; Pollack et al. 1949; Pollack and Wood 1949). Moreover, the muscle pump enhances central venous pressure and thereby increases the stroke volume of the heart (Leyk et al. 1994; Laughlin and Schrage 1999; Rowland 2001). However, in an actual change in body posture from horizontal to upright, gravity also mechanically affects muscle and alters neuromuscular control. Identical exercise performed at horizontal or upright posture may not result in identical muscle action, which complicates the comparison of gravity and posture dependent perfusion effects on muscle. We, therefore, developed a simplified model by combining an electrically driven and robotically controlled leg press with a lower body negative pressure (LBNP) chamber for studying effects exclusively evoked by blood shifts to the exercising leg muscle without any further effects linked with an actual change in posture.

LBNP has been extensively used as means of simulating the cardiovascular and physiological effects of gravity. In light of the upward fluid shift occurring in astronauts during space flight and the beneficial effects of LBNP combined with exercise (Hargens et al. 2013), as well as its successful use in simulating orthostasis, LBNP has been a topic of interest in the area of space physiology. Combined with supine walking and running exercise on a vertical treadmill, LBNP was able to simulate the physiological reactions, i.e. oxygen consumption and heart rate levels, as well as the ground reaction forces generated during upright gait (Boda et al. 2000). In addition, it has also been shown to enhance performance during incremental-load supine dynamic leg exercise until exhaustion and to be a suitable model for upright exercise (Eiken 1988). LBNP has been used to assess the function of the cardiovascular system prior to and after space flight (Baisch et al. 2000) and has been investigated as a possible countermeasure for microgravity-induced deconditioning. Microgravity and its ground-based model HDT bed rest are both known to reduce exercise fitness. Daily supine LBNP treadmill exercise was shown to maintain key exercise fitness parameters including peak oxygen consumption at pre-HDT-bed rest-levels (Watenpaugh et al. 2000), supporting the concept of combining LBNP with exercise countermeasures against deconditioning under long-term microgravity (Murthy et al. 1994).

Depending on the applied LBNP level, its systemic effects have been shown to include a decrease in central venous pressure with a concomitant reduction in stroke volume (SV) leading to a diminished cardiac output. This is almost compensated for by a directly ensuing increase in heart rate (HR) and accompanied by a slow rise in total peripheral resistance stabilizing blood pressure (Berdeaux et al. 1992). LBNP is also known to enhance the local perfusion pressure and consequently the muscle blood flow in the lower extremities (Eiken 1988). Its effects on relaxed leg muscles include an increase in tissue haemoglobin index (Bartels et al. 2011) and deoxygenated blood, predominantly indicating venous blood pooling (Blaber et al. 2013). Superimposed on intense dynamic exercise, LBNP has previously been shown to significantly enhance the level of total and oxygenated haemoglobin in the calf muscle and improve fatigue resistance (Zange et al. 2008). Our current work was focused on studying these effects of LBNP when combined with slow concentric–eccentric resistance exercise. Resistance exercise not only prevents atrophy of chronically unloaded lower limb muscles, but also has the potential of promoting their hypertrophy (Tesch et al. 2004). Given the advantages of combining exercise with LBNP (Boda et al. 2000; Eiken 1988; Hargens et al. 2013; Zange et al. 2008), resistance exercise with LBNP might prove beneficial in countermeasure development for future long-duration space flight.

High muscle forces tend to lead to muscle ischemia, whereas periods of relaxation can facilitate muscle perfusion. Enhanced muscle perfusion in turn benefits energy generating oxidative metabolism, enabling the muscle to maintain its energy levels during prolonged activity (Toussaint et al. 1996). Muscle perfusion can also be enhanced through the muscle pump activity during exercise, depending on the type and frequency of muscle contractions (Laughlin 1987; Sheriff 2003; Sheriff and Hakeman 2001). Low-intensity resistance exercise with slow motion has for instance been shown to have a facilitating effect by enhancing basal femoral blood flow (Tanimoto et al. 2009). Reduced contraction velocity can also enhance adaptive responses to strength training (Schuenke et al. 2012). Our aim was, therefore, to investigate the acute systemic cardiovascular as well as local physiological reactions to LBNP combined with a novel mode of intense, resistive leg press exercise incorporating pauses between contractions. Our exercise protocol included a varying muscle load with the leg press exercise conducted in a slow motion and with a gradually increasing force during extension. These features might have additional relevance in terms of countermeasure activities, as it has been previously reported that leg press training with a constant load could not prevent isokinetic strength losses induced by bed rest (Bamman and Caruso 2000).

Contrary to the venous pooling in an inactive muscle, we hypothesized that muscle relaxation between contractions would benefit the blood flow originating from the muscle pump activity. This would be reflected in elevated total haemoglobin levels in the muscle tissue contributed from the arterial side, increasing the availability of oxygen and possibly leading to enhanced oxidative metabolism. That would in turn lead to increased overall oxygen uptake and a reduced rise in post-exercise lactate levels. The resulting improved maintenance of energy levels could ultimately reduce the neuromuscular compensation of muscle fatigue. We expected that our exercise protocol with LBNP results in a lower increment of EMG-amplitude, indicating a lower increase in the number of recruited motor units to achieve the specified force. Finally, we aimed to verify that muscle oxygenation and performance are affected by simulated orthostasis during slow paced resistive exercise. Our experimental design might consequently also serve as a model test in further studies on the role of gravity induced physiological changes in working leg muscle.

## Methods

### Study participants

Nine healthy male subjects (27 ± 5 years, 181 ± 6 cm, 80 ± 7 kg) with similar profiles of moderate recreational activity completed the study. Subjects were fully acquainted with the experimental approach and provided a written informed consent prior to their participation. Approval was issued by the North Rhine Medical Association’s Ethics Committee (Ethikkommission der Ärztekammer Nordrhein, Düsseldorf, approval no. 2013426).

### General study design

The study followed a crossover design testing the acute effects of 15 slow, high load, concentric and eccentric contractions of knee extensor muscles on a robotically controlled leg press (RCL) under four different conditions in which the following variables were either introduced or omitted: lower body negative pressure (LBNP) as means of substituting orthostasis and 4 s pauses enabling prolonged periods of very low muscle force and muscle perfusion between the contraction intervals. These four conditions were varied in a balanced permutated sequence with a minimal resting period of 7 days between different exercise sessions to avoid any interaction effects (Table 1). Exercise sessions were preceded by familiarization with the RCL and the 1-repetition maximum (1-RM) determination.

**Table 1:** Sequence of exercise sessions

Subject no.	CE	CE + LBNP	EP	EP + LBNP
1	1	2	3	4
2	4	3	2	1
3	3	4	2	1
4	3	4	1	2
5	4	1	3	2
6	2	1	4	3
7	1	4	2	3
8	3	2	1	4
9	1	2	4	3

Varying exercise conditions were permutated

*CE* continuous exercise, *EP* exercise with 4 s long pauses between repetitions, *LBNP* lower body negative pressure

Subjects were asked to follow certain guidelines during their participation in the study, in particular not to change the nature or intensity of their physical activity during the study period. Based on interviews preceding the study, a profile of weekly physical activity (type and duration of exercise) was recorded for each subject. The subjects were advised to maintain their usual pattern of activity and not engage in more strenuous or additional exercise. Extensive physical activity (e.g. intense sports) was not allowed for up to two days prior to each session, to ensure that the subjects were well rested when attending their study appointments. Furthermore, subjects were advised to abstain from alcohol for 24 h prior to their appointments, as well as to eat 3 h beforehand and thereafter only consume water, to ensure an overall comparable metabolic state of the subjects during the sessions. As caffeine withdrawal can negatively impact the ability to perform, we decided against caffeine restriction and rather advised our subjects to retain their usual coffee intake. To maintain sufficient energy levels, subjects were also asked to consume a protein energy drink (Fresubin, Fresenius Kabi) 2 h prior to their exercise sessions. All the above-mentioned points were followed-up on with each subject throughout their participation in the study. Subjects were introduced to these guidelines through an orientation before the study started. They additionally received all the details as handouts and were timely reminded of specific due points by phone or e-mail.

Due to a risk of syncope during the sessions with LBNP, blood pressure and heart rate were monitored for pre-syncopal signs by an independent physician using ECG (Datex-Ohmeda, GE, Germany).

### The robotically controlled leg press with an LBNP chamber (RCL)

**Fig. 1:**
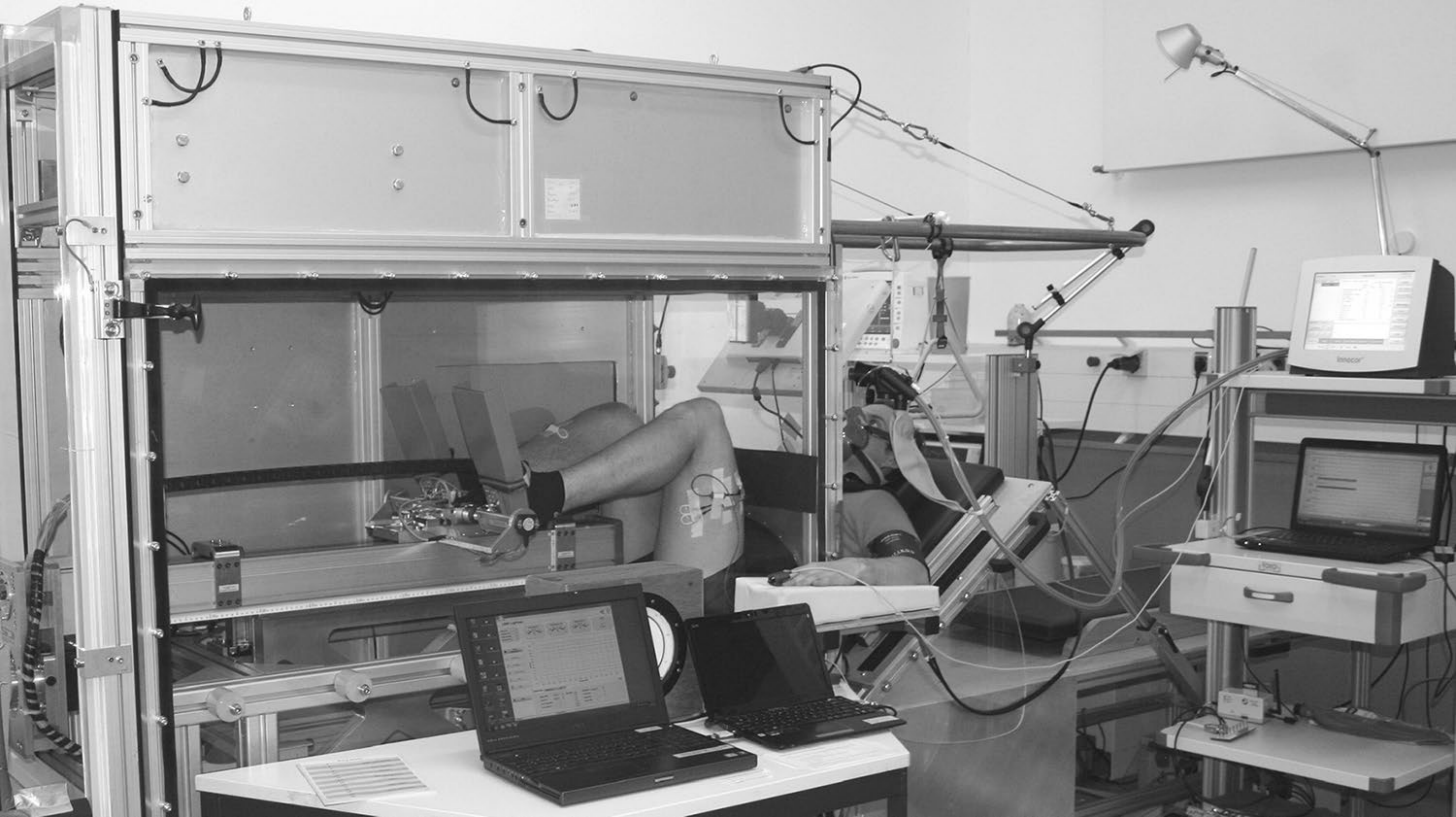
Robotically controlled leg press within the LBNP chamber (Institute of Aerospace Medicine, German Aerospace Center, Cologne)

RCL was developed at the German Aerospace Center (DLR Cologne, Germany) in cooperation with the companies Sensodrive G.m.b.H (Weßling, Germany) and S.E.A. Datentechnik GmbH (Troisdorf, Germany). The linear drive of the leg press is controlled by an electromotor enabling safe exercise without storage of energy by weights or springs. The force–distance profile is freely programmable and allows customized training adapted to individual requirements. The leg press is located within an LBNP chamber that previously contained a treadmill (Watenpaugh et al. 2000). To maintain a stable negative pressure inside the chamber, subjects wore a neoprene suit with the seal placed around their hips (Fig. 1). Subjects rested their upper body on a backrest outside the LBNP-chamber, elevated at a 30° angle, and placed their feet on a pair of pedals horizontally moved by the linear drive, whose height is adjusted to the length of the subject’s legs.

During horizontal movements within the individual range of motion, the knee angle varied between 80° and 125° allowing almost horizontally orientated forces during exercise. Feet were placed on pedals with rotational axes located under the tibia so that foot plantar- and dorsi-flexors were not loaded during exercise. Since subjects were asked to keep a neutral foot angle and maintain low exercise velocity with equal performance from both legs, the foot angles and difference in forces of both feet, as well as the target vs. actual position of the legs were displayed on a screen providing visual feedback. LBNP was set at 40 ± 1 mmHg, starting and ending with a ramp of 4 mmHg/s.

The force–distance profile was designed as gradually increasing, with 10% of the target force applied at knee flexion, reaching the full target force at the middle of the individual range of motion, which then remained constant till the terminal point of extension. Consequently, forces were reversed during eccentric motion. Based on experience, we chose a low force instead of no force for the beginning and the end of a repetition. This low force helped the subjects in controlling the slow velocity of motion at low forces during exercise and maintaining the position of their feet during pauses at knee flexion. Target force for the warm-up was 20% 1-RM, and for the main exercise set 60% 1-RM. Force and distance were continuously measured and used to assess adherence to the exercise protocol.

### Familiarization and 1-RM determination

During familiarization, subjects were acquainted through standardized procedure with the measurements and exercise protocol to be employed during the study. Familiarization was followed by a 30-min break and subsequent 1-RM determination, which consisted of warm-up on the cycle ergometer, two warm-up sets of 15 repetitions on the RCL with a force corresponding to 50% BW separated by 1 min pause, continuing with 3 min pause and a main set at 230% BW, in which the subjects performed as many repetitions as possible. Number of repetitions in the main set was used to calculate the 1-RM based on the Wathan equation (LeSuer et al. 1997), which was subsequently used to determine the target force for the exercise sessions.

### Exercise sessions

The exercise sessions started with a general warm-up on the bicycle ergometer (5 min, 75 W, 80 rpm), directly followed by a 5 min baseline data collection phase on the RCL (Table 2). In the control exercise sessions, the baseline phase was then followed by an additional exercise-specific warm-up. In the LBNP sessions, the initial baseline was followed by a further 8 min baseline phase with 40 mmHg LBNP, enabling the necessary accommodation of the cardiovascular system. The first baseline was designated as BL1 and the baseline directly preceding the exercise-specific warm-up as BL2. Warm-up included two sets of fifteen contractions with 20% 1-RM separated by 1 min pause, followed by a 3 min resting period and the main exercise set (E) comprised of fifteen contractions (C1–C15) with a maximum load corresponding to 60% 1-RM. Depending on the type of exercise, the second warm-up and the main exercise set were accordingly performed in a continuous motion, without pause or intermittently, with 4 s pause during flexion. With the added variation of the presence of LBNP, the study included a total of four different conditions (control/LBNP, without or with 4 s pause). Repetitions were performed at a standardized velocity of 4 s for the concentric and 4 s for the eccentric contraction phase, supported by a metronome and vocal instructions, and by monitoring the actual and the target foot position on the RCL display. Concentric knee extension started at 80° knee angle with a force corresponding to 10% of the target force. Linearly increasing, the force then reached its target level midway through the range of motion, afterwards remaining constant till the terminal point of extension at a 125° knee angle. Force–distance profile was consequently reversed during the subsequent eccentric motion. Main exercise set was followed by a 20 min recovery consisting of two parts: first 5 min of the recovery (R1) occurred with LBNP and the remaining 15 min (R2) proceeded without LBNP. Although the control sessions were conducted solely under ambient pressure, their recovery phases were also subdivided into R1 and R2 for easier comparability with the corresponding LBNP-sessions. Table 2 provides an outline of the exercise sessions.

**Table 2:** Outline of distinct phases during the exercise sessions

Phase	Duration (min)	Contractions	Target force (% 1-RM)	LBNP
1st Baseline, BL1	5	–	–	Off
2nd Baseline, BL2	8	–	–	On
Warm-up, set 1	2	15	6	On
Pause	1	–	–	On
Warm-up, set 2	2 or 3	15	6	On
Pause	3	–	–	On
Main exercise set, E	2 or 3	15	60	On
Recovery 1, R1	5	–	–	On
Recovery 2, R2	15	–	–	Off

Duration of the warm-up and the main exercise set: 2 min, without pause during flexion and 3 min, with 4 s breaks during flexion, respectively

Sum force and foot position were continuously recorded together with the electromyogram data at a sampling rate of 1500 Hz. Analysis was performed for the peak test force (*N*) determined as the average force over 2 s around the turning point in extension, mean test force (*N*) averaged over the entire 8 s contraction interval, single direction range of motion (mm), mean velocity (mm/s) of concentric and eccentric contractions, as well as the concentric and eccentric mean power (W, power = force × velocity × 0.001).

### Near infrared spectroscopy (NIRS)

The concentrations of oxygenated and deoxygenated haemoglobin (O_2_Hb, HHb in µmol/l tissue) were measured with near infrared spectroscopy (NIRS) using the telemetric Portamon device (Artinis Medical Systems BV, Elst, The Netherlands), placed on the right leg, over the belly of the vastus lateralis muscle. Portamon is a small wireless device weighing 75 g, carrying one light sensor and three light sources with emission at wavelengths of 760 nm and 850 nm. Their distance towards the light sensor is 30, 35, and 40 mm, respectively. The proportion of light absorption by the skin is reduced to a minimum by an almost 1.5 mm elevation of the emitters and the receiver over the surface of the device. This elevation reduces thickness and blood content of the light-transmitting portion of the skin. Motion artifacts were minimized by a tight fixation of the device with black elastic kinesiology tape. NIRS signal was recorded continuously at a rate of 10 Hz. During data collection, the device automatically calculated the total haemoglobin content (tHb = O_2_Hb + HHb, µmol/l) and the tissue oxygen saturation index (TSI = 100 × O_2_Hb/tHb, %). BL1 and BL2 mean values were calculated for a 30 s period, from 2 to 1.5 min preceding the directly following phases. O_2_Hb and tHb minima occurred during the high load phases of the contraction intervals in the main exercise set (*E*), with corresponding maxima emerging during the low load phases, i.e., the transitions from one contraction interval to the next. During intermittent exercise, the maxima occurred in the 4 s pauses between the contractions. This recurrent pattern of minima and maxima gave a more precise reflection of the blood filling and Hb oxygenation levels than the corresponding mean values. Therefore, minima and maxima of tHb, O_2_Hb, HHb and TSI were determined for each contraction interval (C1–C15). As in case of the baseline values, the recovery values were also calculated as means during the 30 s period from the onset of the last 2 min of each phase (3–3.5 min and 18–18.5 min into the recovery for R1 and R2, respectively).

### Electromyography

Noraxon Myosystem 1400 (Velamed, Germany) was used for electromyography (EMG), with electrode pairs placed on the vastus lateralis and the biceps femoris muscle, respectively. In case of the right leg, the electrodes were placed distally from the Portamon device. EMG was recorded at 1500 Hz sampling rate throughout the main exercise set. The root mean square values of amplitudes were calculated for the concentric phases of each of the 15 contraction intervals and normalized to the initial values of the first contraction. Corresponding median frequency values are also presented.

### Cardiovascular parameters

Blood pressure was continuously measured using a plethysmographic device (Finometer MIDI, Finapres Medical Systems, The Netherlands) with the finger cuff placed on the fourth finger of the right hand. Based on the blood pressure values, the BeatScope software (ADInstruments, Australia) calculated beat-by-beat the following parameters: heart rate (HR, 1/min), stroke volume (SV, ml), cardiac output (CO, l/min) and total peripheral resistance (TPR, dyn × s × cm^− 5^). For the baseline and recovery phases (BL1, BL2, R1, R2), mean values were calculated as previously described for the NIRS parameters. Mean values were also determined for each contraction interval of the main exercise set (C1–C15).

### Respiratory oxygen uptake and CO_2_ release

Spirometry was performed using the Innocor device (Innovision, Denmark). Oxygen uptake (V′O_2_, l/min), carbon dioxide release (V′CO_2_, l/min), and respiratory exchange ratio (RER = V′CO_2_/V′O_2_) were determined in a breath-by-breath mode. Mean values were calculated for the last 2 min of baseline without LBNP, the entire main exercise set (E), as well as for the first 5 min and the final 15 min of recovery (R1 and R2, respectively). Excess values of V′O_2_ and V′CO_2_ during E, R1 and R2 were determined by subtracting the corresponding baseline values. In addition, a representative time curve of V′O_2_, V′CO_2_ and ventilation (l/min) is presented for the initial recovery (R1 and first 5 min of R2).

### Lactate measurements

Lactate concentration (mmol/l) in capillary blood obtained from the ear lobe was measured with a portable device (Lactate ProTM LT-1710, Arkray) directly after the main exercise set, as well as post 1, 2, 4, 6, 10, 15 and 20 min.

### Perceived exertion

Rating of perceived exertion (RPE) based on Borg‘s Scale with a range of 6–20 (Borg 1998) was determined immediately following the main exercise set.

### Statistical tests

Linear mixed effects models were used to test for significant effects of LBNP and pause, as well as their interactions. Additional tested fixed effects were contraction and side (EMG-amplitude), phase (tHb, O_2_Hb, HHb, TSI, HR, SV, CO, TPR) and time point (lactate). For exercise parameters (force, distance, velocity), only the effect of having four different conditions (without/with LBNP or pause) was tested. Statistical analysis was performed with SPSS (IBM SPSS Statistics Version 21). Data are presented as means ± standard error (SE) or standard deviation (SD), as indicated in the accompanying legends. Statistical significance was rated as *p* < 0.05.

## Results

### Exercise performance

Comparable exercise performance of the knee extensors was accomplished across all four study conditions (Table 3). Peak test force, mean test force, range of motion, concentric and eccentric contraction velocities, as well as the concentric and eccentric power displayed no significant differences between continuous or intermittent exercise under ambient pressure or LBNP, indicating an even implementation of the exercise protocol across the study. No incidence of syncope occurred during exercise with LBNP.

**Table 3:** Summary of the main exercise parameters

Parameter	Exercise sessions				*p*
	No pause		4 s pause		
	Control	LBNP	Control	LBNP	
Test force (*N*)	1037.3 ± 162	1035.9 ± 164.9	1040.1 ± 158.9	1044.6 ± 163.9	0.999
Mean force (*N*)	752.2 ± 128.5	746.9 ± 118.5	784 ± 104.2	766 ± 108.6	0.896
Work distance (mm)	269.7 ± 34.6	264.9 ± 32.8	261.3 ± 32.4	264.1 ± 32.3	0.962
Concentric velocity (mm/s)	67.41 ± 8.66	66.22 ± 8.21	65.32 ± 8.11	66 ± 8.08	0.962
Eccentric velocity (mm/s)	− 67.24 ± 8.58	− 66.04 ± 8.37	− 65.95 ± 7.91	− 65.61 ± 8	0.978
Concentric power (W)	57.19 ± 9.31	56.24 ± 9.5	55.81 ± 9.33	55.96 ± 9.13	0.989
Eccentric power (W)	− 51.17 ± 8.61	− 50.32 ± 8.84	− 50.62 ± 8.57	− 50.37 ± 8.59	0.996

Listed are the absolute values for the main parameters characterizing the exercise protocol (test and mean force, work distance, concentric and eccentric velocity and power). Test force is measured at the turning point between extension and flexion ± 1 s. Values are means ± SD, *n* = 9; *p* values (LME) close to 1 indicate homogenous exercise performance under all four conditions

### Cardiovascular reactions to resistive exercise with LBNP

**Fig. 2:**
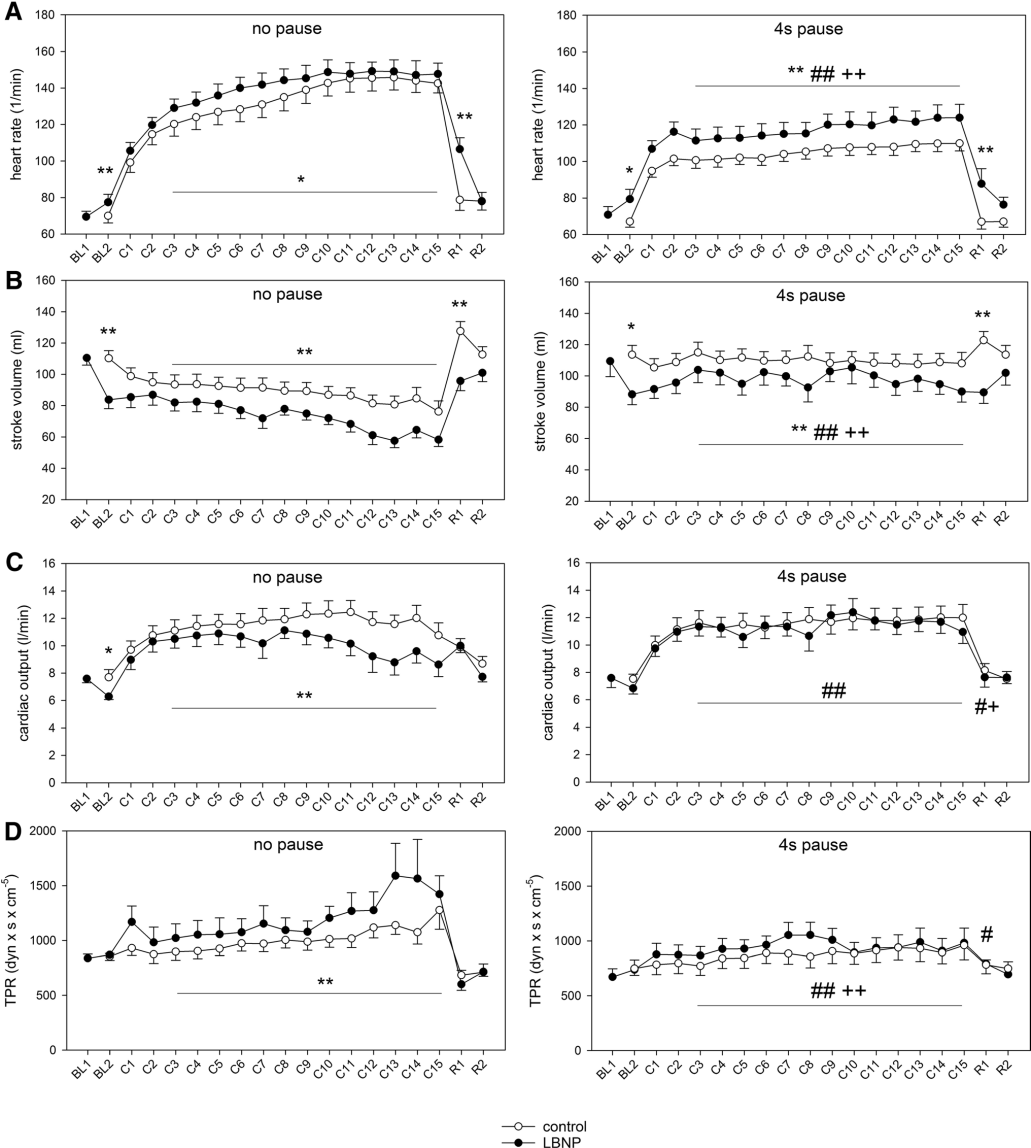
Mean values (± SEM, *n* = 9) of heart rate (**a**), stroke volume (**b**), cardiac output (**c**) and total peripheral resistance (TPR, **d**) at baseline before LBNP onset (BL1, LBNP experiments only), baseline before the first warm-up (BL2), fifteen repetitions of contractions during the main exercise set (C1–C15), after 3 min recovery (R1, when applicable under LBNP) and after 18 min recovery (R2). Exercise sessions were performed under control (open circle) and LNBP (filled circle) conditions, without or with 4 s pause in between repetitions. Statistical significance (*p* < 0.05 or *p* < 0.01) is shown for effects of LBNP during exercise with/without pause (asterisk) and the effects of added pause during exercise under LBNP (hash) or ambient pressure (plus)

During baseline, LBNP led to a moderate orthostatic reaction characterised by an increase in heart rate (HR) from around 70 to 80/min (*p* < 0.05) and a decrease in stroke volume (SV) from approximately 110 to 80 ml (Fig. 2; *p* < 0.01). The resulting cardiac output (CO) decreased from approximately 8 to 6 l/min (Fig. 2; *p* < 0.05). Total peripheral resistance (TPR) was not significantly influenced by LBNP at rest (Fig. 2). During exercise under ambient pressure (control condition), HR and CO increased to approximately 145/min and 12 l/min, respectively. SV moderately decreased to approximately 85 ml and TPR maintained almost stable values. LBNP further augmented the increase in HR during intermittent exercise, as well as the decrease in SV and increase in TPR during continuous exercise (Fig. 2; *p* < 0.01). During the initial recovery with LBNP (R1), directly following the main exercise set, HR decreased in all conditions, but still remained higher with LBNP than under corresponding control conditions and was accompanied by a reduction in SV with LBNP (Fig. 2). In the late recovery phase (R2), HR and SV from the LBNP sessions were reduced to the levels of the corresponding controls. CO and TPR values were not significantly influenced by LBNP during recovery (Fig. 2).

### Haemoglobin content and tissue oxygen saturation

Total haemoglobin (tHb) content was significantly elevated during baseline with LBNP (BL2) by approximately 20 µmol/l (*p* < 0.01). This increase can be attributed to elevated deoxyhaemoglobin (HHb; *p* < 0.01), since oxyhaemoglobin (O_2_Hb) levels were not significantly impacted. Consequently, a slight (under 5%) but significant decrease of the tissue oxygen saturation index (TSI) was observed (*p* < 0.01). The values of tHb, O_2_Hb, HHb and TSI relative to the initial baseline (BL1) did not significantly differ between the exercise sessions.

**Fig. 3:**
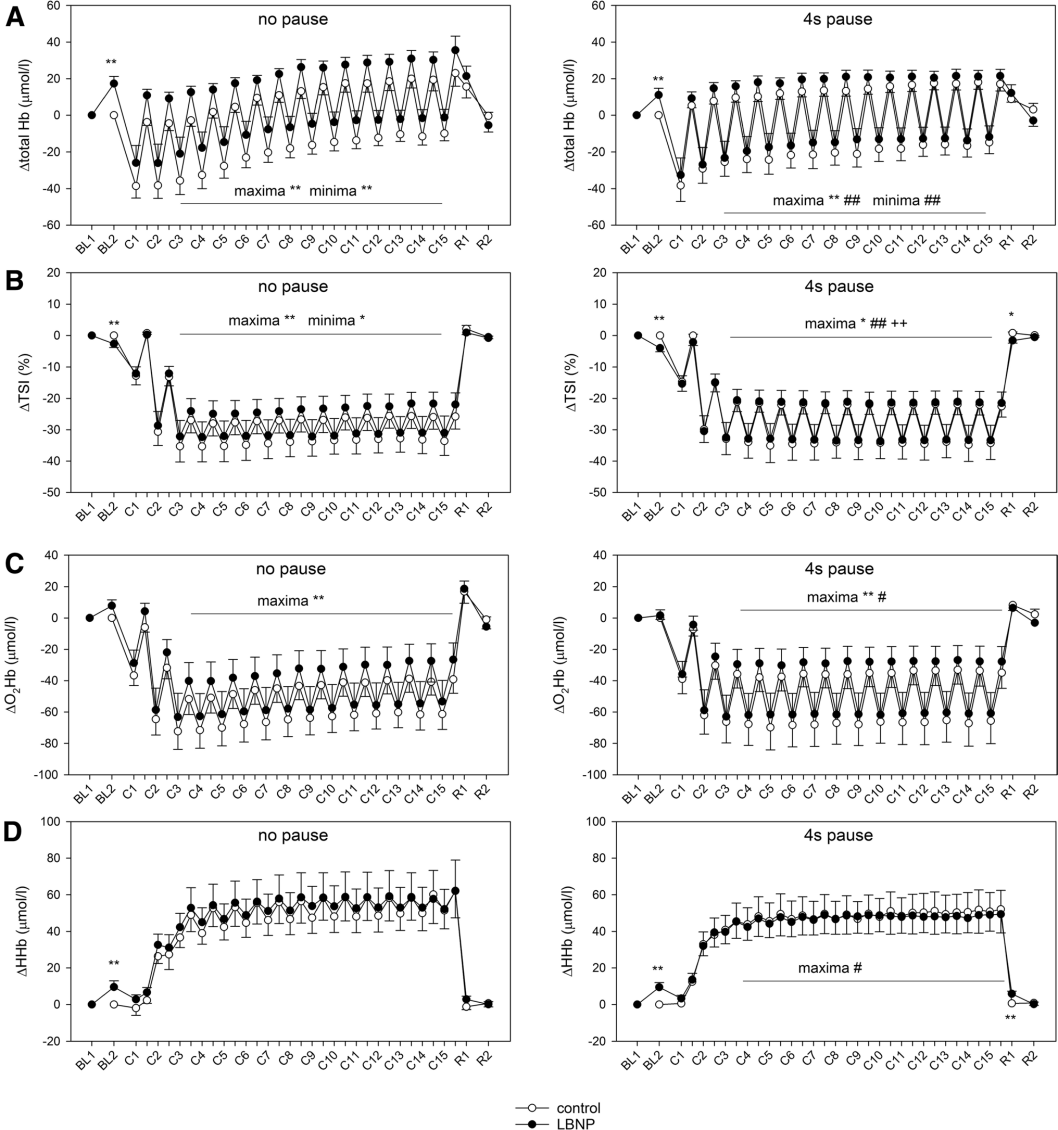
Changes in the levels of total haemoglobin (ΔtHb, **a**), tissue oxygen saturation (ΔTSI%, **b**), oxyhaemoglobin (ΔO_2_Hb, **c**) and deoxyhaemoglobin (ΔHHb, **d**). Time points and statistical significance are labeled as in Fig. 2

During the subsequent exercise set (E), a pattern of minimal values occurring in the periods of high force and maximum values arising in the low force periods of the muscle contraction intervals (C1–C15) was observed for all NIRS-parameters, reflecting the stop and permission of blood flow and the activity of the muscle pump. Maxima of tHb were significantly elevated with LBNP (Fig. 3; *p* < 0.01), more evidently during continuous than during intermittent exercise (approximately 20 µmol/l versus approximately 5 µmol/l difference, respectively). On the other hand, tHb minima were only significantly elevated during continuous exercise with LBNP, by around 10 µmol/l (*p* < 0.01). Likewise, TSI maxima were elevated in both LBNP exercise sessions by a margin of under 5% (*p* < 0.01 for continuous and *p* < 0.05 for intermittent exercise), whereas the TSI minima were only enhanced during continuous exercise, by a similar margin (*p* < 0.05). Maxima of O_2_Hb were elevated with LBNP, both during continuous exercise (*p* < 0.05), as well as during intermittent exercise (*p* < 0.001) by approximately 10–15 µmol/l, with no significant changes in O_2_Hb minima.

Relative tHb and O_2_Hb levels normalized to the corresponding values during BL1 were not significantly influenced by LBNP during the initial or late recovery phase (R1 and R2, respectively), indicating a restoration of the pre-exercise tHb and O_2_Hb concentration. During R1 following intermittent exercise, TSI was slightly decreased with LBNP (under 5%; *p* < 0.05), accompanied by a small increase in HHb (approximately 5 µmol/l). This initial post-exercise response also receded before R2.

### Respiratory oxygen uptake, CO_2_ release and the respiratory exchange rate

**Fig. 4:**
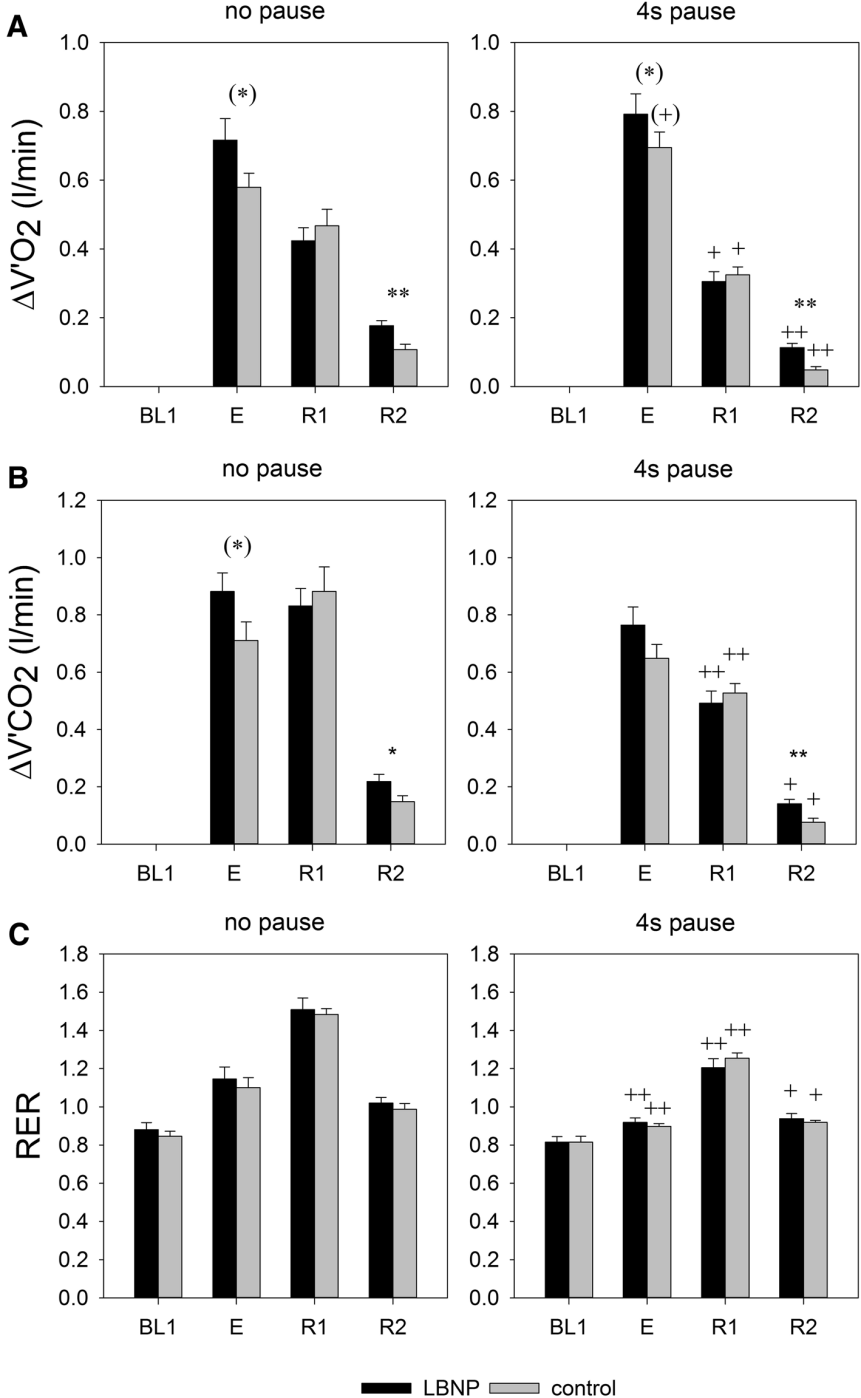
Respiratory oxygen uptake (ΔV′O_2_, **a** means ± SEM, *n* = 9, deltas from baseline 1), carbon dioxide release (ΔV′CO_2_, **b**), and the respiratory exchange ratio (RER, **c**) during exercise sessions (E) and subsequent recovery phases (R1, R2) under control (gray bars) and LBNP conditions (black bars). Statistical significance is labeled as in Fig. 2

**Fig. 5:**
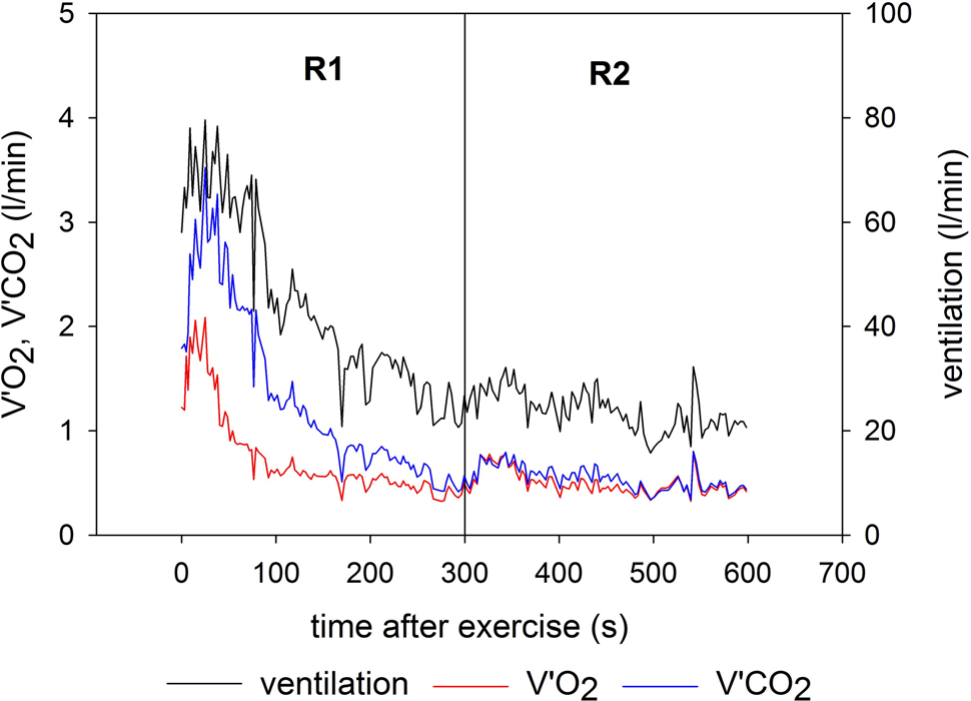
Respiratory oxygen uptake (V′O_2_), carbon dioxide release (V′CO_2_) and ventilation during the recovery stages R1 (with LBNP) and R2 (ambient pressure) following the main exercise set

Excess V′O_2_ and V′CO_2_ were significantly elevated with LBNP during the main exercise set (Fig. 4; V′O_2_, *p* < 0.05, V′CO_2_, *p* < 0.05) and the late recovery phase (V′O_2_, *p* < 0.01; V′CO_2_, *p* < 0.01) compared to the corresponding control interventions, both without and with 4 s pause during flexion. No significant effect of LBNP on excess V′O_2_ and V′CO_2_ or RER was found in the initial recovery phase immediately prior to switching off LBNP. Intermittent exercise led to significantly diminished excess V′O_2_ and V′CO_2_ during the recovery phases under both control and LBNP conditions relative to sessions with continuous exercise, accompanied by an overall reduction in the respiratory exchange ratio (RER; Fig. 4). The shut-down of LBNP during recovery from exercise induced a transient short increase in both V′O_2_ and V′CO_2_ noticeable in the beginning of R2 (Fig. 5). Subsequently, both values decreased to resting levels. This short-term effect following the shut-down of LBNP led to significantly higher values of ∆V′O_2_ and ∆V′CO_2_ compared to the control conditions during R2 (Fig. 4). Overall, no significant influence of LBNP on RER was found.

### Lactate levels in capillary blood from the ear lobe

**Fig. 6:**
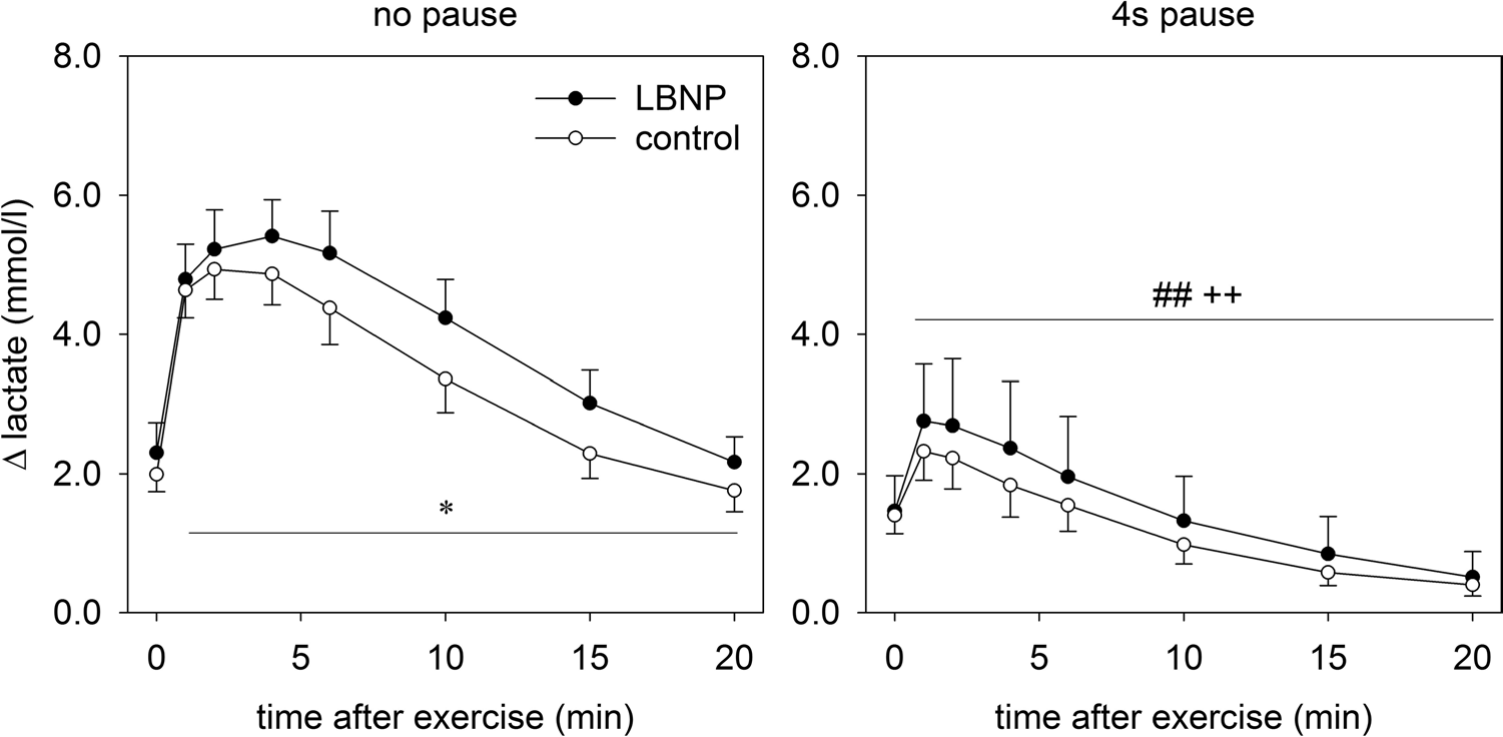
Lactate levels (means ± SEM, *n* = 9, deltas from baseline 1) after the main exercise set, measured in capillary blood obtained from the ear lobe. Statistical significance is labeled as in Fig. 2

Lactate levels increased during the initial 4 min after continuous exercise and 1 min following intermittent exercise. They then gradually returned to baseline values during the overall 20 min recovery period. The observed increase was higher after continuous compared to intermittent exercise (Fig. 6; *p* < 0.01). Lactate was only significantly elevated through LBNP after continuous exercise (*p* < 0.05).

### EMG amplitude and median frequency

**Fig. 7:**
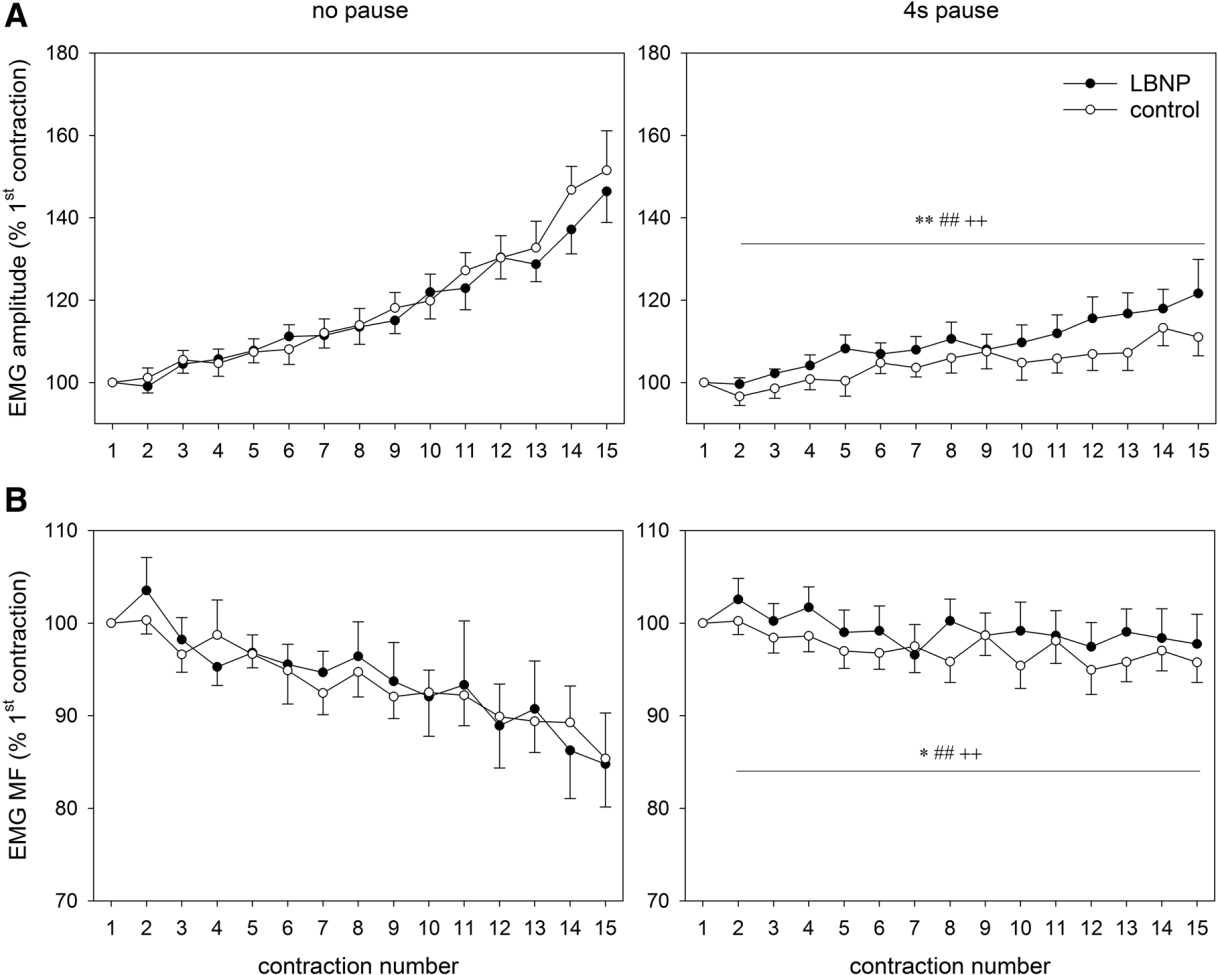
EMG amplitude and median frequency across the fifteen contractions of the main exercise set relative to the first contraction (means ± SEM, *n* = 9), measured bilaterally on the vastus lateralis muscle. Statistical significance is labeled as in Fig. 2

In the vastus lateralis muscle, a gradual increase in the EMG-amplitude was observed during the main exercise set relative to the amplitude of the first contraction. Bilateral measurements showed no significant difference between the data obtained from the left and right leg. The data were, therefore, combined during further analysis (Fig. 7). Rise in the EMG-amplitude of the vastus lateralis muscle was more pronounced during continuous as compared to intermittent exercise, accompanied by a corresponding reduction of the median frequency relative to the first contraction. During continuous exercise, no significant influence of LBNP was found both on the EMG amplitude or median frequency. The EMG-amplitude increase was significantly higher during intermittent exercise with LBNP (*p* < 0.01, Fig. 7). The corresponding median frequency showed a slight decline throughout the exercise session, the values being overall higher under LBNP. The absolute voltage of the EMG amplitude of the biceps femoris muscle was only about 12% of the signal from the vastus lateralis muscle. In continuous exercise, the EMG amplitude of the biceps femoris muscle increased by about 82 ± 48%. During exercise with pauses, the EMG amplitude only increased by about 28 ± 19%. LBNP had no significant effect on the EMG amplitude of the biceps femoris muscle.

### Rating of perceived exertion

After the main exercise set, subjects provided following ratings of perceived exertion (RPE) based on Borg’s scale (Borg 1998): for exercise without pause 17.6 ± 1.6 (mean ± SD) under control conditions and 17.7 ± 1.7 under LBNP; for intermittent exercise with 4 s pause during flexion 16.1 ± 1.6 under control conditions and 15.7 ± 2.1 under LBNP. Rating of perceived exertion was lower for intermittent compared to continuous exercise (*p* < 0.01), with no significant influence of LBNP.

## Discussion

Through our present work, we introduce a novel combination of a robotically controlled leg press within an LBNP chamber as an experimental model with promising results for simulating gravity like blood shifts in exercising leg muscles. Combined with an appropriate experimental design, this unique device enables studying the effects of simulated orthostasis on leg muscle perfusion without additional mechanical and neuromuscular effects linked with an actual change of body posture. In accordance with our introduced hypotheses, we were able to demonstrate the existence of a gravity-dependent muscle pump during slow paced high intensity resistive exercise. Our results demonstrate that LBNP superimposed on a slow concentric–eccentric resistive exercise can enhance blood volume and oxygenation of the knee extensor muscles, suggesting enhanced muscle perfusion.

LBNP at 40 mmHg represents a reductive experimental model that simulates one component of upright posture, namely the fluid shift towards the lower body (Boda et al. 2000; Baisch et al. 2000). In the gravitational field of the Earth, an actual switch from horizontal to upright body posture would result in a more complex pattern of physiological reactions, because of the vestibular sensing and the different biomechanical situation with complex consequences on cardio-vascular and neuro-muscular control. LBNP works independent from gravity. Since the pulling force exerted by LBNP on the subjects’ body was in our study fully compensated by a saddle the subjects occupied, we were able to investigate specifically the effects of the fluid shift at the given exercise conditions. Insofar this study provides a reductive approach on the physiological role of orthostatic fluid shift on muscle oxygenation and metabolism. For our research team, this study is also a step in a program of testing and understanding whether LBNP can improve the effectiveness of strength training in the microgravity environment of a spaceship.

Our present results pertaining continuous exercise without pause during flexion support previous findings, which showed LBNP to reduce SV and CO levels (Eiken 1988) and attenuate the exercise-induced increase in CO during incremental load dynamic leg exercise (Eiken 1987). Prolonged periods of low muscle force through pauses during flexion extended the duration of muscle perfusion, leading to higher CO and lower TPR levels with LBNP compared with continuous exercise and abolishing the difference towards corresponding control conditions. The observed increase in resting heart rate (HR) has also been previously reported with LBNP (Nishiyasu et al. 1999). In contrast to our findings, in this previous study the initial rise in HR during dynamic leg exercise with LBNP was shown to stabilize, whereas HR under control pressure was not reaching a steady state but was continuously increasing at a low rate (Nishiyasu et al. 1999). These different findings could arise from unique patterns of exercise including different power and LBNP levels (50 mmHg in the study by Nishiyasu et al. 1999).

LBNP-induced blood volume shift has previously been substantiated through a decreased tissue haemoglobin index (THI) in the forearm with a concomitant THI elevation in the calf muscle measured with NIRS (Bartels et al. 2011). Our observations regarding enhanced total- and oxygenated haemoglobin content are consistent with previous findings (Hachiya et al. 2004; Nishiyasu et al. 1999; Zange et al. 2008). Since resistive leg exercise reduces the oxygen saturation of the muscle tissue (Downs et al. 2014), the here reported increase in the TSI is likely generated by the superimposed LBNP. Increased blood flow and a recovery of muscle oxygenation during resting periods after exercise (Downs et al. 2014) explain the rise in the TSI during intermittent exercise under ambient pressure to the levels in the corresponding LBNP exercise, whereby in the latter case, maximal filling capacity of the blood vessels was most probably reached.

During exercise with pauses during flexion, V′O_2_ as an indicator of metabolic power was somewhat higher than during continuous exercise, although the pauses reduced the mechanical power because identical work was done over a longer time period. During the recovery phase, V′O_2_ was higher after continuous exercise indicating a higher oxygen debt compared to intermittent exercise. Moreover, RER values higher than 1 during continuous exercise indicate conversion of bicarbonate to CO_2_, likely indicating a ventilatory response to the production of protons by anaerobic glycolysis or an increased ventilatory drive due to strenuous exercise. During recovery from exercise RER always increased above 1 indicating a slower exchange of CO_2_ than O_2_ in the lung and a release of excess CO_2_ formed by buffering processes in the blood. Additionally, continuous exercise resulted in higher RER values during recovery, which support the conclusion on a larger fraction of anaerobic energy metabolism during exercise. In both exercise modes we observed a trend of LBNP promoting higher V′O_2_. Moreover, the differences in V′O_2_ and in V′CO_2_ found at late recovery (R2) indicate that LBNP mediated a pooling of CO_2_-rich and oxygen-poor blood in the lower body that was released after LBNP shut-down and subsequently transported headwards, passing the lung. This finding is supported by the initial brief increase in V′O_2_ and in V′CO_2_ directly upon LBNP shut-down followed by a recovery of the values, indicating an initial transport of O_2_-diminished blood from the lower extremities into the lung rather than a sudden boost of the local energy metabolism in the recovering muscle. This distinct difference in the transport of blood gasses caused by LBNP decreases the comparability of respiratory values during exercise with and without LBNP at least outside long-term steady state periods.

Lower increase of oxygen uptake and a lower rise in lactate levels were previously observed during incremental load dynamic leg exercise with LBNP (Eiken 1988), in contrast to our findings which indicate higher oxygen uptake and lactate levels upon continuous resistive leg press exercise with LBNP. This somewhat counter-intuitive finding could at least in part be attributed to the higher frequency and lower loading of leg muscles during cycling in comparison with our slow paced high intensity resistive exercise on a leg press (Eiken 1988). Compared to biking, during our slow and high force leg press exercise oxidative metabolism may further be limited by contraction-induced inhibition of blood flow and oxygen delivery, which can in part be compensated by anaerobic metabolism. Furthermore, the slow exercise rhythm (4 s into extension and 4 s back into flexion) with a gradually increasing force during extension might also have benefited the lactate washout and hence the detection of higher lactate levels in capillary blood during the current study. Pauses might have allowed the lactate washout to start in smaller increments early on during intermittent exercise, accounting for the overall lower and comparable lactate levels between the control and LBNP condition. Since capillary blood lactate levels were found to correlate well with the muscle lactate content upon leg press exercise (Gorostiaga et al. 2014), our findings suggest that anaerobic metabolism also contributes to balancing the ATP demand during short and intense resistive exercise. However, lactate measured in blood obtained from the ear lobe does only qualitatively reflect anaerobic glycolysis in the working muscle, because of delays in lactate transport and the consumption of lactate in other parts of the body.

Postural effects on the development of fatigue in lower limb muscles manifest at moderate to very high forces with regard to intermittent contractions (Egaña and Green 2007), which is a range that corresponds well to the target force in the present study (60% 1-RM). Contrary to these findings, we observed somewhat higher EMG amplitude levels of the vastus lateralis muscle during intermittent exercise with LBNP versus the corresponding control condition. Beyond the differences in the training design (inclined limb position at 67° angle vs. 40 mmHg LBNP in the present study), a reason for this could also be the duration of the pauses, which was in previous studies twice as long as the duration of contraction and is equivalent to the contraction interval in the present study (Egaña and Green 2007). In the vastus lateralis muscle, the observed higher EMG-amplitude increment during intermittent exercise with LBNP indicates increased activation of motor units compensating for muscle fatigue (Zange et al. 2003), accompanied by a corresponding decline in median frequency. This might be associated with reduced energy content due to diminished oxygenation of the muscle tissue, whose values were approaching those of the corresponding control intervention. In continuous exercise with LBNP, a tendency towards a lower EMG-amplitude increment was becoming apparent in the vastus lateralis muscle. This finding indicates a possible delayed onset of the LBNP effect under the present setting, which could potentially manifest more clearly in a prolonged experiment. The decline of the median frequency was in this case comparable between LBNP and control, however. The voltage of the EMG amplitude in the biceps femoris muscle indicated a rather low co-contraction of this antagonist during concentric and eccentric knee extension exercise. The increasing amplitudes of the antagonists during exercise likely resulted from an increasing co-activation in response to the compensatory increasing activation of the intensively working knee extensor muscles without reflecting the effects of LBNP on knee extensors. Since in this study only one muscle head was measured as an example in both knee extensor and knee flexor musculature, we cannot exclude effects of LBNP on further neuro-muscular adaptation mechanisms, for example, in intermuscular coordination.

The increasing EMG-amplitude of the vastus lateralis muscle was consistent with higher values for the rating of perceived exertion (RPE). The RPE values did not significantly differ between the LBNP and their corresponding control sessions, suggesting a comparable intensity of exercise within individual training protocols. Furthermore, the average test force at the turning point between extension and flexion (± 1 s), mean force, as well as average work distance on the leg press and the concentric and eccentric velocity and power were all comparable between the four tested conditions (without/with LBNP and 4 s pause during flexion), eliminating the hypothetical influence of an uneven mechanical performance on the reported findings on motor unit activation. This is further reinforced by equivalent EMG signal patterns obtained through bilateral measurements, which suggested that the exercise was performed with equal contribution from both limbs.

In summary, we report an exercise protocol design involving a novel, robotically controlled leg press that enables even performance during intense resistive exercise, with added benefit of enhanced muscle perfusion through superimposed LBNP. This combination could ultimately facilitate muscle performance through increased oxygenation and energy provision, delaying the development of muscle fatigue. This is reflected in the enhanced total and oxyhaemoglobin content, and enhanced tissue oxygen saturation index of the knee extensor muscles. Concomitant increases in the overall oxygen uptake and CO_2_ release indicate enhanced oxygen utilization in the working muscle. Furthermore, an ensuing tendency towards reduced motor unit recruitment suggests a possible benefit of LBNP in delaying muscle fatigue. Our findings indicate that LBNP is effective when ischemic periods by high muscle forces are preceded by equal periods of lower forces allowing blood flow through the muscle. Four seconds longer recovery periods between contractions allow full reflow of blood in all cases without an additional benefit of LBNP.

Combining LBNP and resistive exercise has shown promising results and potential benefits for diverse applications, spanning from advancing training techniques on Earth to countermeasure development for long-duration space flight. Due to the advantages of resistive exercise for muscle mass enhancement (Tesch et al. 2004) and strength development, especially one involving concentric and eccentric actions (Dudley et al. 1991), and considering its combination with LBNP offers notable benefits, continued research in this area could provide a meaningful contribution to science. The reported experimental design may serve as a useful model test in further studies on the role of gravity-induced physiological changes in working leg muscle.

## Abbreviations

BL1: Baseline 1 (study phase)
BL2: Baseline 2 (study phase)
BW: Body weight
CO: Cardiac output
E: Exercise (study phase)
ECG: Electrocardiogram
EMG: Electromyography
LBNP: Lower body negative pressure
LME: Linear mixed effects model
Hb: Haemoglobin
HDT: Head-down tilt
HHb: Deoxyhaemoglobin
HR: Heart rate
NIRS: Near infrared spectroscopy
O_2_Hb: Oxyhaemoglobin
R1: Recovery 1 (study phase)
R2: Recovery 2 (study phase)
RCL: Robotically controlled leg press
RER: Respiratory exchange ratio
RPE: Rating of perceived exertion
rpm: Revolutions per minute
SD: Standard deviation
SE: Standard error
SEM: Standard error of the mean
SV: Stroke volume
tHb: Total haemoglobin
TPR: Total peripheral resistance
TSI: Tissue saturation index
V′O_2_: Oxygen uptake
V′CO_2_: Carbon dioxide release
1-RM: 1-repetition maximum
